# Molecular Trajectory of *BRCA1* and *BRCA2* Mutations

**DOI:** 10.3389/fonc.2020.00361

**Published:** 2020-03-25

**Authors:** Yuichiro Hatano, Maho Tamada, Mikiko Matsuo, Akira Hara

**Affiliations:** Department of Tumor Pathology, Gifu University Graduate School of Medicine, Gifu, Japan

**Keywords:** breast, ovary, pancreas, prostate, *BRCA1*, *BRCA2*, cancer predisposition gene, mutational signature

## Abstract

Every cancer carries genomic mutations. Although almost all these mutations arise after fertilization, a minimal count of cancer predisposition mutations are already present at the time of genesis of germ cells. Of the cancer predisposition genes identified to date, *BRCA1* and *BRCA2* have been determined to be associated with hereditary breast and ovarian cancer syndrome. Such cancer predisposition genes have recently been attracting attention owing to the emergence of molecular genetics, thus, affecting the strategy of cancer prevention, diagnostics, and therapeutics. In this review, we summarize the molecular significance of these two *BRCA* genes. First, we provide a brief history of *BRCA*1 and *BRCA2*, including their identification as cancer predisposition genes and recognition as members in the Fanconi anemia pathway. Next, we describe the molecular function and interaction of BRCA proteins, and thereafter, describe the patterns of *BRCA* dysfunction. Subsequently, we present emerging evidence on mutational signatures to determine the effects of *BRCA* disorders on the mutational process in cancer cells. Currently, *BRCA* genes serve as principal targets for clinical molecular oncology, be they germline or sporadic mutations. Moreover, comprehensive cancer genome analyses enable us to not only recognize the current status of the known cancer driver gene mutations but also divulge the past mutational processes and predict the future biological behavior of cancer through the molecular trajectory of genomic alterations.

## Introduction

Cancer cells harbor several genetic mutations and epigenetic modifications, which are believed to have arisen from sequential and multistage neoplastic processes (1). However, the mechanism of cellular transformation remains unclear because each oncogenic event is broken down into a molecular reaction, which seems to occur stochastically and independently during oncogenic events in each cancer case. Even if comprehensive genomic data are available, it is still difficult to determine the correct order of genomic alteration.

A possible breakthrough in the understanding of the evolutional process of cancer cells *in vivo* was provided by studies conducted on the hereditary cancer syndrome, which is due to a germline mutation of the cancer predisposition gene (2, 3). Before the establishment of molecular evidence, clinicians had insights into the familial breast cancer (4). Subsequently, genetic and reverse-genetic research revealed the initial and the following steps in the neoplastic process, which has contributed to novel strategies for cancer prevention, diagnostics, and therapeutics.

The main purpose of this review is to summarize the molecular biology associated with the representative cancer predisposition genes, *BRCA1* and *BRCA2*, and to speculate on the missing link between normal and cancer cells.

## Discovery of *BRCA1* and *BRCA2*

Cancer predisposition genes, *BRCA1* and *BRCA2*, were first discovered in the genetic study on familial breast cancer (5) (Table 1). At that time, linkage analyses with DNA polymorphic markers were detecting the causal relationships between certain genetic diseases and specific genomic loci (6). Similarly, a variable number tandem repeat marker known as *D17S74*, revealed that the candidate familial breast cancer gene is located at chromosome 17q21 (7). Thereafter, this locus, also called the “breast cancer, early onset,” or *BRCA1*, was indexed for the comprehensive genetic disease database, Mendelian inheritance in Man (MIM), and was given the reference number 113705 (8). After the inter-laboratory competition over 4 years (9), positional cloning of *BRCA1* was first achieved using an emerging technique which required the use of bacterial artificial chromosomes (10). In contrast, the second breast cancer predisposition gene, *BRCA2*, was discovered at chromosome 13q12 by other DNA polymorphic markers, *D13S260*, and *DS13S263* (11), and registered with the MIM number 600185. The discovery of the second breast cancer predisposing gene was followed by the *BRCA1* cloning, and subsequently, the race to clone *BRCA2* was completed the following year by the same research team (12).

**Table 1:** Summary of *BRCA1* and *BRCA2*.

	***BRCA1***	***BRCA2***
Location	Chromosome 17q21	Chromosome 13q12
Functional domains (their main binding partners)	RING domain (BARD1) Coiled coil domain (PALB2) BRCT domain (ABRA1, CtIP, and BRIP1)	Eight BRC repeats (RAD51) DNA binding domain (DSS1)
Synonym as FA genes	*FANCS*	*FANCD1*
Cardinal function as a cancer predisposition gene	Homologous recombination	Homologous recombination
Association between promoter methylation and silencing	Established	Not established
Reversion of the mutated gene	Sometimes	Sometimes
Mutational signatures associated	Signature 3 or SBS3/ID6	Signature 3 or SBS3/ID6
Association with breast cancer	Basal-like and/or triple negative breast tumor, high grade histology	Lobular neoplasia, moderate to high grade histology
Association with ovarian cancer	High-grade serous carcinoma, SET-type	High-grade serous carcinoma, SET-type Possibly clear cell carcinoma
Association with pancreatic cancer	Not established	Rarely, high grade histology
Association with prostate cancer	Not established	Rarely, high grade histology

*RING, really interesting new gene; BARD1, BRCA1-associated RING domain protein; PALB2, partner and localizer of BRCA2; BRCT, BRCA1 C terminus; ABRA1, abraxas; CtIP, CtBP interactive protein; CtBP, C-terminal binding protein; BRIP1, BRCA1-interacting protein C-terminal helicase 1; DSS1, deleted in split-hand/split foot protein 1; FA, Fanconi anemia; SBS, Single base substitution; ID, Small insertion and deletion; SET, solid, pseudoendometrioid, and transitional cell carcinoma-like histology*.

## Functional Similarities and Differences Between *BRCA1* and *BRCA2*

Both *BRCA1* and *BRCA2* are large genes, which consist of ~100 and 70 kb, respectively; the largest exon of both the *BRCA* genes is exon 11. Although these genetic features resemble the proof of breast and ovarian cancer predisposing gene family at the first glance, there is no homology between *BRCA1* and *BRCA2* (13). *BRCA1* contains a nuclear localization sequence (NLS) and three functional domains; RING, coiled coil, and BRCT domains interact with the BRCA1-associated RING domain protein (BARD1), the partner and localizer of BRCA2 (PALB2), and several other proteins that include abraxas (ABRA1), CtBP interactive protein (CtIP), and BRCA1-interacting protein C-terminal helicase 1 (BRIP1), respectively (13). These interactions lead to versatile functions of BRCA1: DNA damage sensing, cell cycling regulation, E3 ubiquitin ligase activity, chromatin remodeling, and homologous recombination (HR). In contrast, *BRCA2* has NLS, eight BRC repeats (14), and a DNA binding domain. Unlike BRCA1, the functional domains of BRCA2 are principally associated with the HR-related proteins, including RAD51 and deleted in split-hand/split foot protein 1 (DSS1) (15, 16). Therefore, the unique molecular traits of each BRCA protein create a difference between *BRCA1*- and *BRCA2*-mutated cancers.

As a common function between *BRCA1* and *BRCA2*, HR is an essential DNA repair system that enables the error-free recovery of double strand breaks (DSBs) (17). DSBs are the most severe DNA damage, the accumulation of which results in genetic translocation and cell death (18). In the condition of homologous recombination deficiency (HRD) by BRCA dysfunction, restoration of DSBs depends on an error-prone repair machinery, known as non-homologous end joining (NHEJ). Such an HRD, also called genomic instability, is advantageous for the progression of *BRCA*-associated cancer to effectively gain sequence and structural variance, especially in the early phase.

## Other Inherited Breast and Ovarian Cancer Genes Aside From *BRCA1* and *BRCA2*

Because *BRCA1* and *BRCA2* account for ~25% of the familial breast and ovarian cancers (19), this section describes other breast and ovarian cancer predisposition genes. A linkage analysis study revealed that the third candidate hereditary breast cancer gene, *BRCA3*, was suspected at the *BRCA2* neighboring locus, 13q21-22, in intact *BRCA1*/*BRCA2* Nordic cohorts (20); however, the replication study failed to demonstrate the cancer susceptibility (21). These findings suggest that the current genetics-based research has been unable to identify the next cancer predisposition gene or that all *BRCA* genes have already been found.

Another technique to identify novel breast and ovarian cancer genes is to identify a gene cluster, such as *BRCA* genes, that play a role in the DNA repair system. Remarkably, HR is related to the Fanconi anemia (FA) pathway, which mediates repair of the interstrand crosslink (ICL) (22). FA is an inherited hematopoietic disorder that gives rise to myelodysplastic syndrome and leukemia. To date, over 20 genes have been identified as FA predisposing genes, and the germline mutation of the *FANCA* gene accounts for approximately two-thirds of FA cases (23). Most of the FA genes play an important role in the formation of the FA core complex, which binds at the ICL site and then activates the downstream signaling to repair this severely damaged DNA. Finally, the damaged sequence is removed by HR. Therefore, the defective FA pathway leads to cancer predisposition through genetic instability, such as BRCA1 and BRCA2 dysfunction.

Considering the functional significance of HR in the FA pathway, *BRCA1* and *BRCA2* have been refocused as FA susceptibility genes. Of the eight FA genes detected, *FANCD1* was identified as *BRCA2* (24). In contrast, *BRCA1* has been recently recognized as *FANCS* (25). However, germline mutations of *BRCA* genes lead to bone marrow failure less frequently than mutations in other FA genes, likely because they are absent from the FA core complex.

Individuals with germline mutations of the FA genes are susceptible not only to hematopoietic but also to solid malignancies. Multiple gene panel studies have revealed that inherited breast and ovarian cancers rarely harbor germline mutations of FA genes, including *BRIP1/FANCJ, PALB2/FANCN*, and *RAD51C/FANCO* (26). Based on the latest National Comprehensive Cancer Network Guideline (27), *PALB2* is categorized as a gene associated with breast cancer risk, whereas *BRIP1* and *RAD51C* are categorized as genes associated with ovarian cancer risk.

The remaining clinically significant, inherited breast and ovarian cancer genes are the so-called cancer predisposition genes: *ATM, CDH1, CHEK2*, mismatch repair genes, *NBN, NF1, PTEN, RAD51D, STK11*, and *TP53* (27). Therefore, an investigation of these cancer predisposition genes is effective in detecting the pathogenic allele in the case of the non-*BRCA* inherited breast and ovarian cancer.

## Significance of *BRCA* Mutations

Although numerous germline *BRCA* mutations, also called sequence variants, have been reported to date, not all the variants lead to predisposition to cancer. Therefore, interpretation of the clinical significance of the detected mutation is a challenge in medical practice. To determine whether the detected sequence variant is pathogenic or not, the American College of Medical Genetics and Genomics (ACMG), together with the Association for Molecular Pathology and the College of American Pathologist, issued the revised universal guidelines for the interpretation of sequence variants (28). Based on the evidence of pathogenicity or benignity, this guideline classifies the sequence variants into five categories: pathogenic, likely pathogenic, uncertain significance, likely benign, and benign. In practice, pathogenic and likely pathogenic variants require further medical management, whereas other variants do not require such intervention. Nevertheless, variants of uncertain significance, which are found in up to 20% of *BRCA1*/*BRCA2* genetic tests (29), need follow-up to monitor the manifestation of the true nature of the variants; e.g., variant reclassification programs. The major databases and platforms that contain information on *BRCA1* and *BRCA2* variants are as follows: BRCA Exchange (30), ClinVar (31), the Human Gene Mutation Database (HGMD) (32), the Leiden Open Variation Database (LOVD) (33), the Consortium of Investigators of Modifiers of BRCA (CIMBA) (34), and the Evidence-based Network for the Interpretation of Germline Mutant Allele (ENIGMA) (35).

Recently, the international collaboration study conducted by CIMBA clarified different cancer risks related to *BRCA* genes (36). Consistent with the previous studies (37–39), both *BRCA1* and *BRCA2* genes contain several cancer risk regions. The ovarian cancer cluster region (OCCR) of both *BRCA1* and *BRCA2* largely overlaps with exon 11, whereas the breast cancer cluster regions (BCCRs) are located on the exterior of exon 11. The mutation in these cancer cluster regions leads to increased cancer risk of the corresponding organ. Additionally, the mutational type of the *BRCA* genes also affects the breast and ovarian cancer risk. Collectively, the diversity of the *BRCA* sequence variants implies not only the general cancer risk but also the specific susceptible organ, and, therefore, the detailed classification of the pathogenic variants would be effective to determine the optimal medical management.

## DNA Methylation Status of the *BRCA* Genes

The dysregulation of the *BRCA* genes arises not only from genetic alternations but also from epigenetic modifications. At the transcriptional level, *BRCA1* is regulated by the DNA methylation status at its upstream CpG island (40–42). Consistent with the promoter hypermethylation, *BRCA1* is silenced in sporadic breast and ovarian cancer (43, 44). The aberrant *BRCA1* promoter methylation is found in approximately one-ninth of ovarian cancer tumors (45–47) and in one-fourth of breast basal-like tumors (48), suggesting that *BRCA1* silencing is considered a leading non-genetic case of *BRCA1* inactivation in sporadic wild-type *BRCA* cancer. The comprehensive ovarian cancer genomic studies revealed that hypermethylated-*BRCA1* ovarian cancer with platinum therapy had a similar prognosis as the intact *BRCA* cancer, whereas *BRCA1*/*BRCA2*-mutated ovarian cancer showed better prognosis than the wild-type cancer (46, 47). On the other hand, cancer with homologous *BRCA1* hypermethylation showed a good response to an emerging therapeutic agent (described in a later section), the PARP inhibitor, which was same as the response of cancer with *BRCA* germline mutation (49). These evidences suggest that quantitative methylation analysis of *BRCA1* promoter would be needed to predict the clinical behavior of hypermethylated *BRCA1* cancer.

Conversely, the functional significance of the nearest CpG islands of *BRCA2* still remains unclear. Unlike *BRCA1, BRCA2* promoter methylation is not considered a leading cause of *BRCA2* dysfunction (45–48, 50). However, the specific CpG site methylation is a possible marker of germline *BRCA* mutations (51). Because functional significance of the aberrant methylation still remains unclear, further investigations would be needed.

## Reversion of the *BRCA* Mutation

Reversion is defined as the secondary mutation of an inherited mutant gene, which restores normal function in somatic cells (52). For example, the pathogenic *BRCA* allele sometimes reverts to the wild-type sequence via an additional point mutation (back mutation) (53, 54). Conversely, additional insertion/deletion of *BRCA* genes amends the altered reading frame normally (in-frame mutations), thus, converting it to the non-pathogenic allele. These genetic alterations are considered to be a late stage oncogenic event to reactivate the HR pathway, and it consequently renders the cancer cells resistance to lethal DNA damage. Interestingly, approximately a quarter to half of ovarian cancers with germline *BRCA1*/*BRCA2* mutations exhibit the reversion of the inherited mutation and chemoresistance after chemotherapy (47, 55, 56), suggesting that *in vivo* retrieval of *BRCA* function is a potent oncogenic event to resist unwanted DNA damage.

## Mutational Signature of *BRCA* Dysfunction

The forthcoming breakthrough in carcinogenesis research is the “mutation signature,” which stands for a unique pattern of genetic alterations in somatic cells. Given that every mutation arises from a specific molecular reaction, the characteristic sets of the genetic alterations are good evidence for mutational processes in cancer cells. This concept enables researchers to convert the vast genomic data on cancer cells into evidence on the current status of cancer-related genes and sheds light on the history of cancer progression.

Owing to the emerging technology, such as next-generation sequencing (57), the first series of comprehensive somatic mutation research successfully demonstrated the close relationship between a certain type of cancer and mutational signatures. Briefly, the melanoma cell line frequently carried C>T and/or CC>TT transition, which is consistent with the effect of ultraviolet light exposure on pyrimidine bases (58). Conversely, the small-cell lung cancer cell line harbored predominantly G>T, G>A, and A>G transitions, which are interpreted as the modification of purine bases by tobacco smoke carcinogens (59). Interestingly, both studies also highlighted the presence of other mutational signatures, suggesting that somatic cells experience multiple mutational processes *in vivo*.

**Figure 1:**
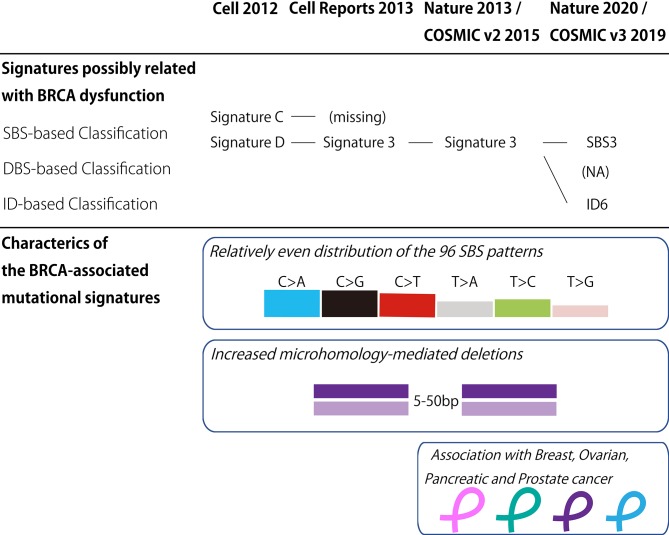
BRCA-associated mutational signature. **(Upper panel)** Classification of the mutational signatures possibly related with BRCA dysfunction. The details of each classification are found in the references 60, 61, 62, and 64. **(Lower panel)** Characteristics of the BRCA-associated mutational signatures. COSMIC, the Catalog of Somatic Mutations in Cancer; v2, version 2; v3, version 3; SBS, Single base substitution; DBS, Double base substitution; ID, Small insertion and deletion; NA, not applicable.

In the last decade, the classification of mutational signatures has rapidly progressed (Figure 1). The classification of mutational signatures was first initiated in a whole-genome study of human breast cancers (60). Owing to the complementation between pyrimidine and purine nucleobases in the double helices, all single base substitutions (also known as point mutations) can be summarized into the following six patterns: C>A/G>T, C>G/G>C, C>T/G>A, T>A/A>T, T>C/A>G, and T>G/A>C transitions. Additionally, to consider the sequence context of the mutated base, these six mutation classes are further subdivided into 96 trinucleotides patterns by referring to the neighboring bases: the 5′- and 3′-base (each base has four types). By analyzing these 96 trinucleotides patterns in 21 different types of breast cancers with mathematical models, five distinctive molecular signatures were extracted. The mutational spectrum of these signatures possibly reflected either aging (spontaneous deamination of 5-methyl-cytosine: Signature A), overexpression of cytidine deaminase belonging to the APOBEC family (Signatures B and E), or *BRCA1*/*BRCA2* mutations (Signatures C and D). Unlike the other mutational signatures, the *BRCA1*/*BRCA2* mutation-associated signatures were unique in regard to the relatively equal distribution of the 96 trinucleotides patterns. Additionally, *BRCA1*/*BRCA2*-mutated cancer carried microhomology-mediated deletions more frequently compared with the wild-type cancers. These genomic abnormalities are likely due to the dysfunction of HR when double strand breaks occur. Subsequently, the additional breast cancer genome study failed to reproduce the Signature C-like pattern; thus, the *BRCA1*/*BRCA2* mutation associated Signatures C and D was combined into Signature 3 (61).

Thereafter, the international collaborative research group analyzed the large collection of somatic mutations for various cancer types to identify further detailed classes of mutational signatures (62). Although this study identified the 21 distinctive patterns of mutational signatures, the etiology remained unknown for approximately half of the mutational signatures. The mutational signature for *BRCA1/BRCA2* mutations, or Signature 3, was reconfirmed in this study, and documented in version 2 of the Catalog of Somatic Mutations in Cancer (COSMIC) mutational signatures (63).

To date, the classification of mutational signatures continues to evolve. Version 3 of COSMIC mutational signatures is composed of three conceptual sets: Single Base Substitution (SBS), Double Base Substitution (DBS), and Small Insertion and Deletion (ID) Signatures (64). This detailed scheme sorts the *BRCA1*/*BRCA2*-associated mutational signature into SBS and ID. In other words, the relatively equal SBS distribution and microhomology-mediated deletions of Signature 3 are interpreted as SBS3 and ID6, respectively.

The analysis of mutational signatures reveals the DNA damage and repair processes of the cancer genome, which arise from the specific molecular reaction. Given the close relationship between Signature 3 and *BRCA* mutations, this genome-wide mutational pattern would be applied to the analysis of cancer genome (50, 65). Increased Signature 3 activity was observed not only in the dysfunction of *BRCA1* and *BRCA2* but also in the inactivation of other HR-related genes, including the *PALB2* germline mutation and *RAD51C* hypermethylation. Remarkably, the increased Signature 3 activity is significantly associated with biallelic mutation, loss of heterozygosity, or epigenetic silencing of the HR-related genes. In contrast, HR-related incomplete inactivation of the gene, e.g., a monoallelic mutation, did not achieve significant Signature 3 enrichment. Therefore, Signature 3 is the circumstantial evidence of HRD, and a good predictor of pathogenic variants of HR-related genes.

## Characteristics of Cancer With *BRCA* Dysfunctions

The link between *BRCA* mutations and specific types of cancer has been emerging. The recent TCGA study addressed the molecular classification of gynecologic and breast cancers, and acknowledged the existence of a subset of cancers with *BRCA*-associated mutational signatures (66). In breast cancer, *BRCA1*-mutated carcinoma is significantly associated with the basal-like subtype that exhibits negative expression of the estrogen receptor (ER), progesterone receptor (PGR), and ERBB2/HER2 (67–69). Additionally, *BRCA1*-mutated and basal-like breast cancer are high grade carcinomas with frequent *TP53* mutations (70, 71), indicating that coexisting *BRCA1* and *TP53* mutations facilitate breast cancer progression. In comparison with *BRCA1*-mutated cancer, *BRCA2*-mutated breast carcinomas frequently express ER and PGR; additionally, HER2 is expressed at the same frequency (69). Furthermore, the histological grade of *BRCA2*-mutated breast carcinoma is generally lower than that of *BRCA1*-mutated breast carcinoma. Regarding the histological type, lobular carcinoma is typically prevalent in the *BRCA2*-carriers, whereas medullary carcinoma is more common in *BRCA1*-carriers.

Lately, in the breast surgical specimens of *BRCA*-carriers, the dedicated histological examination revealed a distinctive pathologic condition known as “hyaline fibrous involution” (72). Lee et al. reported that hyaline fibrous involution was frequently associated with *BRCA*-mutated perimenopausal women. This unusual histological finding, including diffuse thickening of the fibrous band in the benign breast lobule, likely arises from the abnormal DNA repair state in non-neoplastic breast epithelium. Although this atrophic-like alteration is a promising premalignant lesion that is rarely found in the benign breast disease, we believe that hyaline fibrous involution is an unexpected chance to suspect inherited cancer in cases without genetical test and clinical history.

Conversely, ovarian cancer among *BRCA*-carriers tends to be the most frequent histological type; it is a high-grade serous carcinoma (HGSC) (73). HGSC is a representative type II carcinoma (74), which almost always exhibits high grade nuclear atypia arising from *TP53* mutations (46). The prophylactic surgical specimens revealed that the fallopian tube sometimes contained serous intraepithelial carcinoma (STIC) with *TP53* mutations even in asymptomatic *BRCA*-carriers (75, 76). Interestingly, the putative precursor lesion of STIC, or the p53 signature (77), which already carries the *TP53* mutation, is also sometimes found in the fallopian tube, regardless of the *BRCA* genotype. Additionally, the *TP53* mutation type of the p53 signature is occasionally discordant with that of HGSC (78). These findings suggest that the functional significance of *BRCA* mutations is the promotion of neoplastic cells rather than the initiation of minute precursors. Additionally, they suggest that inherited ovarian cancer is most probably an inherited “tubal” cancer, on the basis of the tubal origin theory of HGSC (79).

Notably, HGSC with *BRCA* dysregulations, including *BRCA1*/*BRCA2* mutations and *BRCA1* promoter hypermethylation, are associated with specific morphological “SET” patterns: Solid, pseudoEndometrioid, and Transitional cell carcinoma-like histology (80, 81). Recognition of the SET variant is in line with the recent diagnostic concept for ovarian carcinoma; there are five major histological types that reflect unique molecular characteristics and precursor lesions, and mixed-type ovarian carcinoma accounts for a rare fraction of ovarian epithelial malignancies (82). Although ovarian transitional cell carcinoma was a distinct entity (83), this malignant tumor was incorporated into HGSC in the World Health Organization 2014 classification because of the similarity of the molecular characteristics between the two carcinomas (84). Importantly, SET-type HGSC shows good therapeutic response compared to the conventional-type HGSC, likely due to the HRD arising from *BRCA* dysregulation. Thus, the SET pattern is a diagnostic and therapeutic predictor for HGSC; therefore, pathological examination still remains important in the era of molecular oncology.

Another possible genetic-pathologic correlation between clear cell carcinoma (CCC) and *BRCA2* mutations (85, 86) seems contradictory to the findings of other research groups (87, 88). Because CCC is a type I carcinoma that originates from endometriosis-related cysts or lesions (74), the pathogenesis of CCC is generally unrelated to the above-mentioned high-grade serous carcinogenesis. Nevertheless, three mixed CCC and HGSC cases were reported based on immunohistochemical and genetic analyses (82). The two of the three mixed CCC and HGSC cases were true combined type I and II carcinomas, whereas the remaining case was pure HGSC. These findings suggest that CCC and HGSC might arise from the common precursor cells or that CCC is sometimes misinterpreted as a HGSC by histological assessment only. Therefore, further data are needed to confirm this ovarian genetic-pathologic correlation.

In addition to breast and ovarian cancers, pancreatic and prostate cancers rarely harbor *BRCA* mutations (89, 90) and *BRCA*-associated signatures (62, 64). The clinical sequence studies reveal that the germline *BRCA2* mutation is detected in ~5% of metastatic prostate carcinoma cases (91–93). Histologically, *BRCA2*-mutated prostate carcinoma is associated with high grade histology (94, 95), including ductal (96) and endocrine (97, 98) differentiation. On the other hand, pancreatic cancer also harbors *BRCA2* mutations. Of the common types of cancer, including pancreatic ductal adenocarcinoma (PDAC) and neuroendocrine tumors (PanNET), ~4 and 1% of PDAC and PanNET possess germline *BRCA2* mutations, respectively (99, 100). These findings suggest that mutated *BRCA2*-carriers should exercise caution regarding the development of extra-mammary and uterine adnexal cancers.

## Therapeutic Approach to the *BRCA*-Mutated Cancer

Because breast and ovarian cancer predisposition genes were identified, the principal strategy of hereditary cancer management involves the early detection of cancer by frequent medical checks, including mammogram, breast MRI, transvaginal ultrasound, and serum CA-125 test, frequently referred to as surveillance. In some cases, this entails the surgical removal of the susceptible organs, if deemed medically necessary. Traditionally, pathogenic *BRCA*-carriers require a prophylactic surgery to prevent breast and/or ovarian cancer even in their reproductive age (101). The resected breasts and uterine adnexa contain premalignant lesions and/or microscopic carcinomas (102, 103), which imply the presence of candidates for future malignancy. Indeed, *BRCA*-carriers sometimes suffer from contralateral breast cancer after the first breast cancer. Therefore, bilateral mastectomy is effective to prevent multiple and hererochronous cancer. In addition, in a recent study, it was revealed that oophorectomy slightly assisted in decreasing contralateral breast cancer (104).

Recent molecular and clinical evidence endorses molecular therapy for *BRCA*-mutated cancer. As described previously, *BRCA*-mutated cancer generally exhibits high-grade histology and aggressive phenotypes but responds favorably to platinum-containing chemotherapy (105–108). Such platinum sensitivity is probably due to *BRCA*-associated HRD that fails to recover platinum-induced ICL (109).

A novel molecular treatment using poly (ADP–ribose) polymerase (PARP)-inhibitor is also based on HRD in the *BRCA*-mutated cancer cells. PARP1 is a cardinal DNA repair molecule in the case of single strand breaks (SSB) (110). Inhibition of PARP1 results in the occurrence of DSBs, which is the failure of the replication fork through SSB repair (111, 112), as well as the disturbance of the NHEJ repair pathway, by blocking the chromatin remodeler known as CHD2 (113). Together, PARP1 inhibitor and HRD accumulate the critical DSB damage in the *BRCA*-mutated cancer cells. Consistent with the results of these *in vitro* studies, PARP inhibitors have the effect of suppressing the *BRCA*-mutated cancer regardless of the cancer type (114–117). Currently, clinical use of these promising drugs has been approved by the FDA (118). In the future, genetic testing of the *BRCA* mutation would be necessary to determine the optimal therapeutic plan for individuals with advanced cancer.

## Conclusion

As the molecular functions of *BRCA1* and *BRCA2* have been elucidated, the clinical focus on these cancer predisposition genes shifts toward the development of therapeutic strategies. Additionally, comprehensive cancer genome analysis illustrates not only the present status of cancer related genes but also the past and ongoing mutational processes arising from specific molecular reactions. In the future, the prospective biological behavior of cancer will be predicted via the molecular trajectory of genomic alteration.

## Author Contributions

YH contributed to the conception of the work and wrote the manuscript. MT, MM, and AH contributed to the revisions of the manuscript. All authors have read and approved the submitted manuscript.

### Conflict of Interest

The authors declare that the research was conducted in the absence of any commercial or financial relationships that could be construed as a potential conflict of interest.
